# Prevalence and determinants of fetal macrosomia in Bangladesh

**DOI:** 10.3389/fped.2024.1405442

**Published:** 2024-08-02

**Authors:** Md. Zahidul Islam, Mohammad Rocky Khan Chowdhury, Baki Billah, Mamunur Rashid, Russell Kabir, Mehedi Hasan, Manzur Kader

**Affiliations:** ^1^ Department of Public Health, First Capital University of Bangladesh, Chuadanga, Bangladesh; ^2^ Faculty of Biological Sciences, University of Rajshahi, Rajshahi, Bangladesh; ^3^ Department of Epidemiology and Preventive Medicine, School of Public Health and Preventive Medicine, Monash University, Melbourne, VIC, Australia; ^4^ Department of Public Health and Sports Science, Faculty of Health and Occupational Studies, University of Gävle, Gävle, Sweden; ^5^ School of Allied Health, Faculty of Health, Medicine and Social Care, Anglia Ruskin University, Chelmsford, Essex, United Kingdom; ^6^ School of Science and Technology (SST), Bangladesh Open University (BOU), Dhaka, Bangladesh; ^7^ Department of Medical Science, School of Health and Welfare, Dalarna University, Falun, Sweden

**Keywords:** infant, birth weight, macrosomia, determinants, Bangladesh

## Abstract

**Background:**

Fetal macrosomia, marked by excessive birth weight, is a significant public health issue in developing countries, yet it has received less attention compared to low birth weight. This study aims to determine the prevalence of fetal macrosomia in Bangladesh and its associated factors.

**Methods:**

The study utilized data from 4,754 women with complete birth weight information of their children from the Bangladesh Multiple Indicator Cluster Survey (MICS) −2019, defining fetal macrosomia as newborns with a birth weight ≥4,000 g regardless of gestational age. Bivariate logistic regression assessed associations between independent variables and fetal macrosomia, presenting adjusted odds ratios (AOR) and a 95% confidence interval (CI), while controlling for potential confounders such as women's age, wealth index, education, healthcare utilization, comorbidities, newborn sex, and place of residence.

**Results:**

The prevalence of fetal macrosomia was 11.6%. Significant associations with fetal macrosomia included higher maternal age group (30–34 years) (AOR = 1.36, 95% CI = 1.07–1.74), secondary level of mother's education (AOR = 1.95, 95% CI = 1.43–2.66), experienced physical attacks (AOR = 1.41, 95% CI = 1.06–1.88), hypertension during pregnancy (AOR = 1.54, 95% CI = 1.15–2.07), and rural residence (AOR = 1.25, 95% CI = 1.15–1.49). Female infants had 18% lower odds of being macrosomic compared to male infants (AOR = 0.82, 95% CI = 0.72–0.93).

**Conclusion:**

One in ten infants in Bangladesh are born with macrosomia, necessitating a multi-faceted approach involving improving maternal nutrition, promoting healthy lifestyles, enhancing access to quality prenatal care, and addressing socioeconomic, residential, and healthcare system challenges, underlining the importance of further community-based research to expand the study's scope.

## Introduction

Abnormal birth weight, encompassing both low birth weight (LBW) and high birth weight (fetal macrosomia), plays a crucial role in predicting children's growth, development, and mortality (1, 2). While most research has focused on LBW, there has been a notable global increase of 15%–25% in fetal macrosomia occurrences in recent decades, observed in both developed (5%–20%) and less developed countries (0.5%–15%) (3, 4). Factors contributing to this increase include sedentary lifestyles, imbalanced nutritional practices during pregnancy, higher rates of maternal obesity, gestational diabetes, and changes in social and demographic patterns (3, 5, 6).

Defining fetal macrosomia, characterized by considerably high birth weight, lacks consensus among researchers and obstetricians, with varying thresholds employed in different studies, such as birth weights exceeding 4,000, 4,200, or 4,500 g (7). Nevertheless, most researchers describe fetal macrosomia as a birth weight of 4,000 g or more, regardless of gestational age (2, 8). Fetal macrosomia significantly impacts maternal and neonatal health, contributing to increased infant and child mortality and morbidity rates (9). During pregnancy, the presence of a macrosomic fetus poses risks for both the newborn and mother, including an elevated likelihood of cesarean section, prolonged labor, postpartum hemorrhage, uterine rupture, puerperal infection, anesthetic complications, and the development of type 2 diabetes after pregnancy (3).

Studies on fetal macrosomia remain limited worldwide. In Uruguay, maternal age and obesity were identified as risk factors, while in China, Ethiopia, and Ghana, various maternal (e.g., age, education, BMI, gestational age, diabetes), child-related (sex), and contextual factors (household wealth index) were associated with fetal macrosomia (9–13). However, in many developing countries like Bangladesh, fetal macrosomia and its risk factors are underreported due to lack of quality data.

In Bangladesh, a few hospital-based studies have reported higher fetal macrosomia rates in mothers with diabetes, gestational diabetes, and pre-pregnancy obesity (14–16). These studies, however, are often small-scale and limited to specific hospital settings and cohorts, potentially failing to provide a comprehensive and representative picture of fetal macrosomia prevalence and its determinants in Bangladesh. Thus, this study aims to investigate the prevalence and determinants of fetal macrosomia in Bangladesh using a nationally representative sample.

## Method

### Data source: multiple indicator cluster survey

The Multiple Indicator Cluster Survey (MICS) in Bangladesh is a cross-sectional study that provides nationally representative data. The MICS-2019 aimed to gather comprehensive information on 144 key indicators related to maternal reproductive health, child health, development, nutrition, and demographic profiles of respondents. Data collection occurred through face-to-face interviews using a standardized questionnaire between January 19, 2019, and June 1, 2019. The survey covered all eight administrative divisions of Bangladesh, namely the Southern region (Barisal division), Southeastern region (Chittagong), Central region (Dhaka), Western region (Khulna), Upper-central region (Mymensingh), Midwestern region (Rajshahi), Northwestern region (Rangpur), and Eastern region (Sylhet).’

The MICS utilized a two-stage stratified cluster sampling method to select the survey sample. In the first stage, 64,400 households and 3,220 primary sample units (PSUs) were chosen from Enumeration Areas (EAs) using probability proportional to size techniques. These EAs were systematically listed from the Bangladesh Population and Housing Census 2011, which included 65,193 urban areas and 228,340 rural areas. In the second stage, data were collected from 20 households within each PSU using equal probability systematic sampling. This multistage sampling approach, along with sampling weights, aimed to minimize potential sampling bias. Sample weights were calculated at each sampling stage and adjusted for non-response to obtain final standard weights. Additionally, all ever-married women aged 15–49 years with children aged less than 5 years from the selected households were interviewed without replacement to prevent selection bias. From the 64,000 selected households, a total of 64,378 women aged 15–49 were successfully interviewed. Furthermore, 9,285 women who had at least one live birth in the last two years were initially selected to collect birth weight data. Among them, 4,754 women had complete data on their child's birth weight and were retained for the analysis of this study. Further details can be found in the MICS-2019 report (16).

### Dependent variable

The dependent variable in this study was baby born with fetal macrosomia. In MICS-2019, live births with a reported birth weight were presented based on either a written record, the mother's report, or a combination of both (16).

The definition of macrosomia varies among researchers. According to Boulet et al. (2003), birth weights were categorized as low (<2,500 g), average (≥2,500 g and <4,000 g), grade I (4,000–4,499 g) macrosomia, grade II (4,500–4,999 g) macrosomia, and grade III (over 5,000 g) macrosomia (17) Henriksen (2008) suggested that infants born weighing 4,000 g (4 kg) or above, or falling into grades I, II, and III, were considered macrosomic (11). In this study, infant with macrosomia was considered dependent variable and coded 1 for macrosomia, coded 0 for no macrosomia.

### Independent variables and operational definitions

This study incorporated variables previously identified as significant in the literatures in associating macrosomia (4, 11, 18, 19). Variables including women's characteristics, such as, women's age (in years) (15–19, 20–24, 25–29, 30–35, 35 and above); level of education (no formal education, primary secondary, higher secondary and higher); women experienced infant death (no, yes), experienced physical attack (yes, no), happiness (happy, unhappy), received antenatal care (no, yes), had functional disability (yes, no), hypertension during pregnancy (no, yes), toxoid injection during last pregnancy (no, yes), and children ever born (4 and higher, less than 4); children characteristics, such as, sex of newborn (male, female); and contextual factors including mass media exposure (no, yes), wealth index (poor, middle, rich), and place of residence (urban, rural) (Supplementary Table 1).

### Handling missing data

According to UNICEF and WHO, missing birth weight data can be handled using imputation method (16). Further, the misreporting or heaping cases (e.g., 500 g, 100 g) were replaced by 2,500 g (16) Missing values were imputed using Multiple Imputations by Chained Equations (MICE) (20) MICE has emerged as one of the principal statistical approaches to dealing with missing data, which involves multiple imputations, as opposed to single imputations, in order to account for the statistical uncertainty associated with imputations. The chained equations approach can also handle variables of various types and complexities.

### Statistical analysis

Baseline characteristics of the respondents was assessed using descriptive statistics. The Chi-square test was employed to assess the association between various exposure variables and the occurrence of fetal macrosomia. The comparison of prevalence was considered significant based on significant level at *p* < 0.05 in the Chi-square test. The multivariable logistic regression analysis was carried out to identify the most important variables associated with macrosomia. Variables found significant at the level *p* < 0.25 in the Chi-square test were entered into the multivariable analysis (21) This choice was made to be more inclusive and capture factors that may have important implications, even if their associations were not considered statistically significant at the traditional significant level (*p* < 0.05). Odds ratio was assessed to determine the magnitude and direction of the associations, along with their corresponding Confidence Intervals (CIs). Significant value for multivariable logistic regression was set up at *p* < 0.05. To account for the complex sampling design, including factors such as sampling weight, cluster, and strata, the Stata command “svyset” was employed. This command ensured that the estimates and statistical inferences obtained from the analysis were adjusted to accurately reflect the complex sampling design, enhancing the validity of the findings. Stata version 17 (StataCorp LP, College Station, Texas) was used for entire analyses.

### Ethical consideration

The MICS 2019 was administered by the Bangladesh Bureau of Statistics (BBS) and financially supported by the United Nations Children's Fund (UNICEF), enjoyed additional technical backing from the United Nations Population Fund (UNFPA), the Global MICS team, and the International Centre for Diarrheal Disease Research, Bangladesh (icddr,b). The survey protocol adhered to ethical standards, securing verbal consent from all female participants aged 15–49 prior to their involvement.

The present study employed data from the 2019 Bangladesh MICS, publicly accessible via the designated repository at https://mics.unicef.org/surveys. As the dataset is openly available and obtained through a survey with established ethical protocols, no further ethical clearance was deemed necessary for the current research endeavor.

## Results

### Background characteristics

Around one-third of women (32.2%) were situated within the age bracket of 20–24 years. About 9.2% of mothers who had children had not received any formal education. Hypertension during pregnancy were noted in 75.4% of the women. Of children, slightly over half (51.1%) were male. Approximately 44.1% of the children hailed from a poor socio-economic background, while 78.1% of the total population resided in rural areas. The detailed breakdown is provided in Table 1.

**Table 1:** Background profile of the respondents.

Factors	Frequency	Crude percentage (%)	Weighted percentage (%)	Total/missing
Maternal factors				
Women's age (in years)				
15–19	1,234	13.3	13.6	9,285/0
20–24	2,988	32.2	32.1	
25–29	2,562	27.6	27.5	
30–34	1,640	17.7	17.6	
35 and above	861	9.3	9.3	
Level of education				
No formal education	846	9.1	9.2	9,285/0
Primary	2,151	23.2	23.2	
Secondary	4,691	50.5	50.0	
Higher secondary and higher	1,597	17.2	17.6	
Women experienced infant death				
No	8,328	89.7	89.5	9,285/0
Yes	957	10.3	10.4	
Women experienced physically attacked				
No	8,862	95.4	95.0	9,285/0
Yes	423	4.6	5.0	
Overall happiness				
Happy	9,026	97.2	97.2	9,285/0
Unhappy	259	2.8	2.8	
Received antenatal care				
No	1,678	18.1	17.2	9,285/0
Yes	7,607	81.9	82.8	
Had functional disability				
Yes	106	1.1	1.1	9,285/195
No	8,984	96.8	96.8	
Hypertension during pregnancy				
No	2,304	24.8	24.3	9,285/ 1,678
Yes	6,981	75.2	75.7	
Tetanus toxoid injection during last pregnancy				
No	691	7.4	7.5	9,285/0
Yes	8,594	92.6	92.5	
Children ever born				
4 and higher	1,065	11.5	11.6	9,285/0
Less than 4	8,220	88.5	88.4	
Children characteristics				
Sex of newborn				
Male	4,794	51.6	52.1	9,285/0
Female	4,491	48.4	47.9	
Contextual factors				
Mass media exposure				
No	3,496	37.6	34.7	9,285/0
Yes	5,789	62.3	65.3	
Wealth index				
Poor	4,117	44.3	40.1	9,285/0
Middle	1,810	19.5	19.0	
Rich	3,358	36.2	40.9	
Place of residence				
Urban	1,774	19.1	21.9	9,285/0
Rural	7,511	80.9	78.1	

### Prevalence of fetal macrosomia

**Figure 1:**
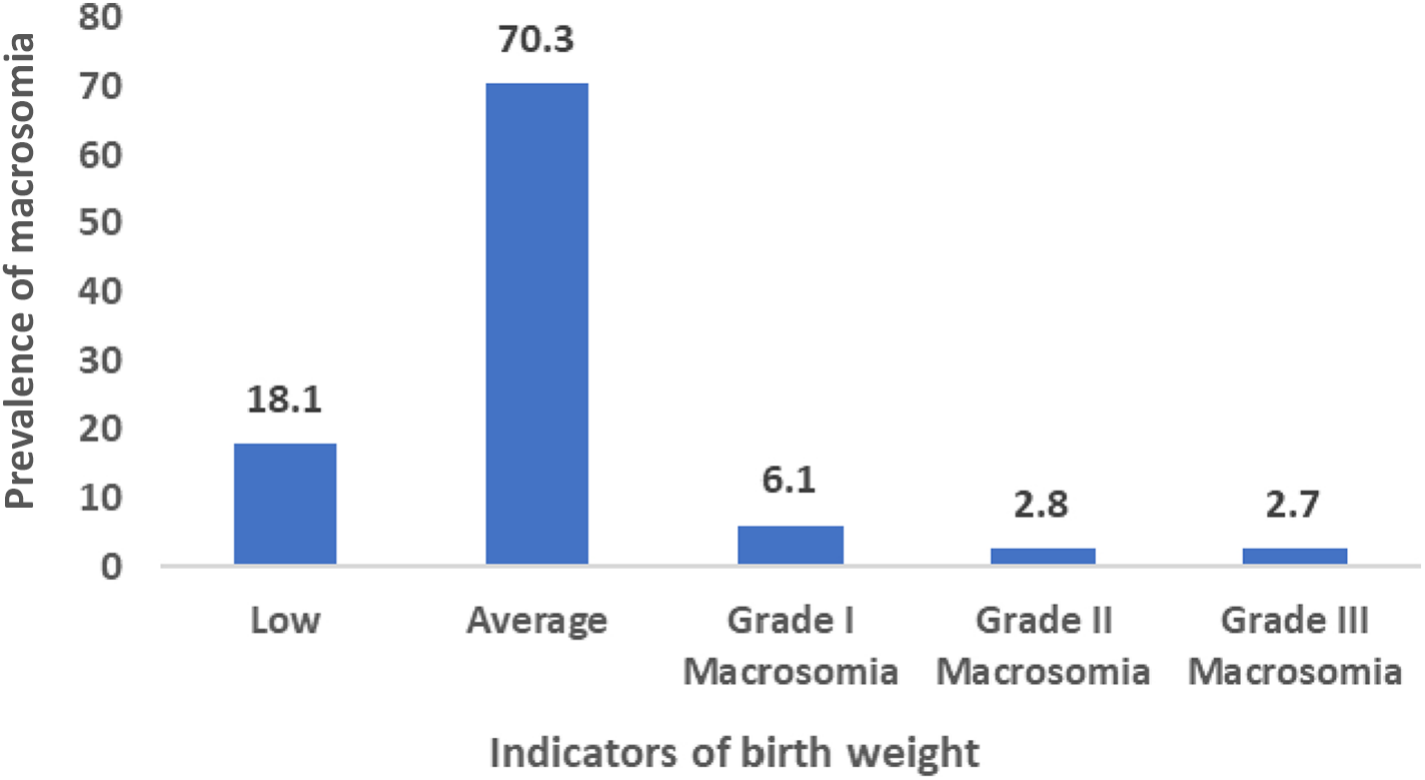
Prevalence of different birth weight infants.

**Figure 2:**
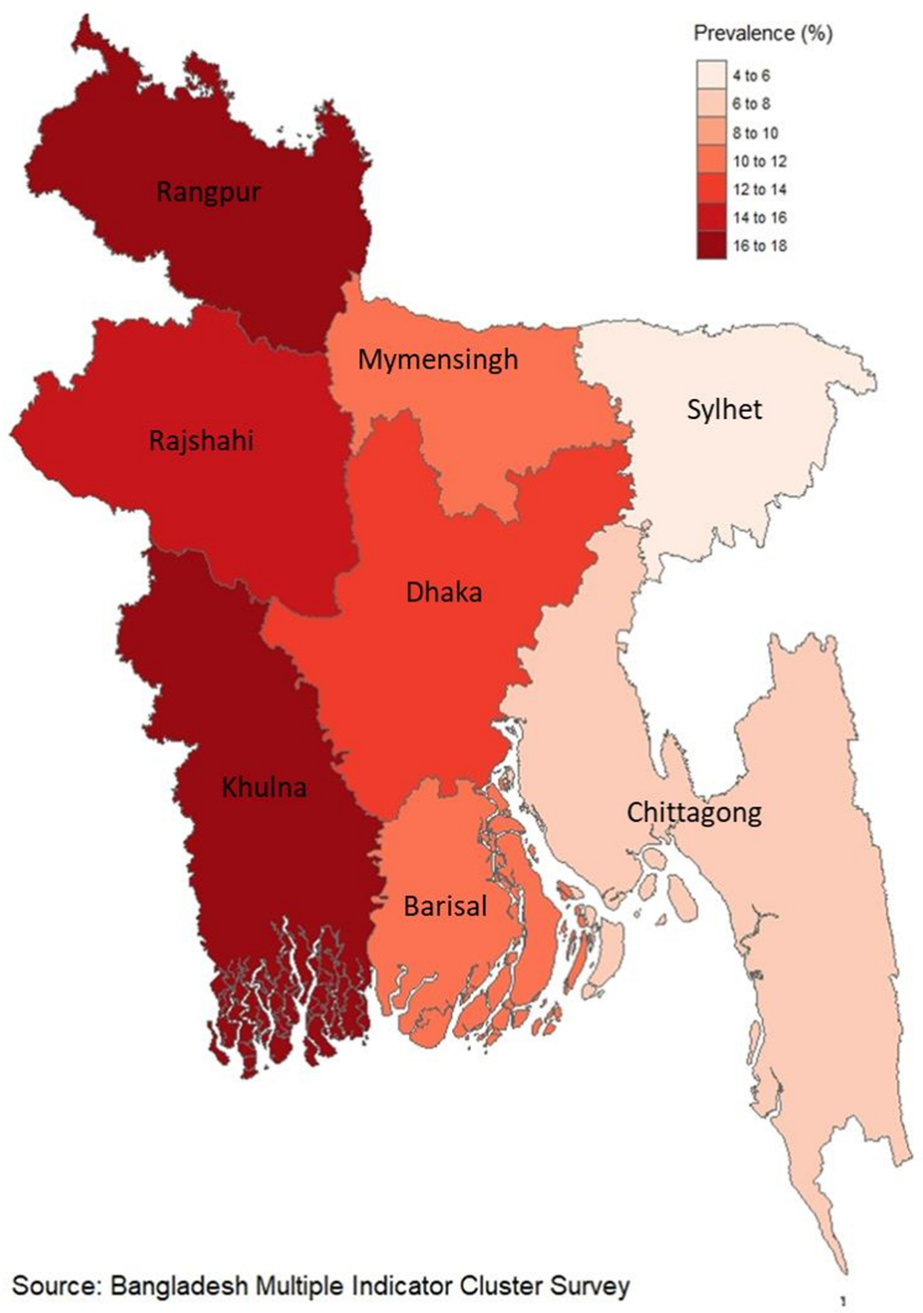
Regional distribution of the prevalence of fetal macrosomia.

In Bangladesh, the occurrence of fetal macrosomia among newborns stands at 11.6%, as depicted in Figure 1. Moreover, within the Khulna division, which encompasses the western region, a significant prevalence of fetal macrosomia is noted at 16.8%, as illustrated in Figure 2.

The prevalence of fetal macrosomia is notably higher among women with a history of gestational hypertension, reaching 12.9%. Similarly, those who have received education beyond primary level, specifically secondary education to higher education, demonstrate comparatively higher rates of fetal macrosomia, recorded at 12.8% and 12.3%, respectively. Furthermore, a substantial increase in the prevalence of fetal macrosomia is observed among women who have undergone antenatal care (12.4%), delivered a male infant as their most recent child (12.4%), and belong to a higher socio-economic status (12.1%), as indicated in Table 2.

**Table 2:** Prevalence of fetal macrosomia.

Variables	Number	Prevalence (95% CI)	*p*-value
Women's age (in years)			
15–19	126	10.4 (8.6, 12.5)	0.092
20–24	382	12.9 (11.6, 14.3)	
25–29	293	10.6 (9.4, 12.0)	
30–34	198	12.2 (10.5, 14.1)	
35 and above	89	10.2 (8.2, 12.6)	
Level of education			
No formal education	53	6.2 (4.6, 8.3)	<0.001
Primary	216	10.6 (9.2, 12.1)	
Secondary	616	12.8 (11.7, 13.9)	
Higher secondary and higher	203	12.3 (10.6, 14.2)	
Women experienced infant death			
No	988	11.6 (10.8, 12.4)	0.198
Yes	100	11.4 (9.2, 13.9)	
Women experienced physically attacked			
No	1,027	11.4 (10.7, 12.1)	0.077
Yes	61	15.1 (11.6, 19.4)	
Overall happiness			
Happy	1,056	11.5 (10.8, 12.3)	0.746
Unhappy	32	12.8 (8.7, 18.3)	
Received antenatal care			
No	128	7.4 (6.1, 9.0)	<0.001
Yes	960	12.4 (11.6, 13.3)	
Had functional disability			
Yes	13	14.5 (8.3, 24.2)	0.868
No	1,055	11.5 (10.8, 12.3)	
Hypertension during pregnancy			
No	181	7.6 (6.4, 8.8)	<0.001
Yes	907	12.9 (12.0, 13.8)	
Tetanus toxoid injection during last pregnancy			
No	65	9.7 (7.4, 12.6)	0.049
Yes	1,023	11.7 (11.0, 12.5)	
Children ever born			
4 and higher	101	10.3 (8.4, 12.5)	0.016
Less than 4	987	11.7 (11.0, 12.5)	
Sex of newborn			
Male	609	12.4 (11.4, 13.5)	0.002
Female	479	10.6 (9.7, 11.7)	
Mass media exposure			
No	367	10.6 (9.5, 11.8)	0.005
Yes	721	12.1 (11.2, 13.0)	
Wealth index			
Poor	437	10.8 (9.8, 12.0)	0.013
Middle	226	12.0 (10.4, 13.8)	
Rich	425	12.1 (10.9, 13.3)	
Place of residence			
Urban	189	10.0 (8.6, 11.6)	0.121
Rural	899	12.0 (11.2, 12.9)	
Total	1,088	11.6 (10.8, 12.3)	

### Determinants

The findings of the study reveal notable associations between various maternal characteristics and the likelihood of fetal macrosomia occurrence. Women aged between 30 and 34 demonstrated a statistically significant 1.36 times higher adjusted odds ratio (AOR) for experiencing fetal macrosomia compared to their counterparts aged 15 to 19 (AOR = 1.36, 95% CI = 1.07–1.74, *p* = 0.013). Similarly, women with secondary education exhibited a substantially elevated AOR of 1.95 (95% CI = 1.43–2.66, *p* < 0.001) for fetal macrosomia in contrast to those lacking formal education. Moreover, a history of physical attacks among women was associated with a 1.4 times higher likelihood (AOR = 1.41, 95% CI = 1.06–1.88, *p* = 0.017) of delivering macrosomic infants. Additionally, mothers with a prior diagnosis of hypertension during pregnancy exhibited a 1.5 times increased probability (AOR = 1.54, 95% CI = 1.15–2.07, *p* = 0.004) of fetal macrosomia occurrence compared to those without such medical history.

Residential setting also emerged as a significant factor, with women residing in rural areas manifesting a 1.25 times higher probability (AOR = 1.25, 95% CI = 1.15–1.49, *p* = 0.015) of delivering macrosomic offspring compared to their urban counterparts. Furthermore, a noteworthy gender disparity was observed, wherein female infants exhibited a 12% decreased likelihood (AOR = 0.82, 95% CI = 0.72–0.93, *p* = 0.003) of being macrosomic in comparison to male infants, as delineated in Table 3.

**Table 3:** Determinants of fetal macrosomia.

Factors	COR (95% CI)	*p*-values	AOR (95% CI)	*p*-values
Women's age (in years)				
15–19	1.00		1.00	
20–24	1.29 (1.04–1.60)	0.020	1.34 (1.07–1.66)	0.009
25–29	1.14 (0.91–1.42)	0.259	1.21 (0.97–1.51)	0.093
30–34	1.21 (0.95–1.53)	0.199	1.36 (1.07–1.74)	0.013
35 and above	1.01 (0.76–1.35)	0.952	1.35 (0.98–1.86)	0.060
Level of education				
No formal education	1.00		1.00	
Primary	1.67 (1.22–2.28)	0.001	1.56 (1.14–2.15)	0.006
Secondary	2.26 (1.69–3.02)	<0.001	1.95 (1.43–2.66)	<0.001
Higher secondary and higher	2.18 (1.59–2.98)	<0.001	1.78 (1.26–2.51)	0.001
Women experienced infant death				
No	1.00		1.00	
Yes	0.87 (0.70–1.08)	0.198	0.95 (0.75–1.19)	0.631
Children ever born				
4 and higher	1.00		1.00	
Less than 4	1.30 (1.05–1.61)	0.016	1.06 (0.81–1.38)	0.646
Women experienced physically attacked				
No	1.00		1.00	
Yes	1.29 (0.97–1.70)	0.078	1.41 (1.06–1.88)	0.017
Received antenatal care				
No	1.00		1.00	
Yes	1.75 (1.44–2.12)	<0.001	1.05 (0.75–1.47)	0.794
Hypertension during pregnancy				
No	1.00		1.00	
Yes	1.75 (1.48–2.07)	<0.001	1.54 (1.15–2.07)	0.004
Tetanus toxoid injection during last pregnancy				
No	1.00		1.00	
Yes	1.30 (1.00–1.69)	0.050	1.11 (0.85–1.45)	0.436
Sex of newborn				
Male	1.00		1.00	
Female	0.82 (0.72–0.93)	0.002	0.82 (0.72–0.93)	0.003
Mass media exposure				
No	1.00		1.00	
Yes	1.21 (1.06–1.39)	0.005	1.06 (0.92–1.23)	0.409
Wealth index				
Poor	1.00		1.00	
Middle	1.20 (1.01–1.43)	0.035	1.04 (0.87–1.25)	0.648
Rich	1.22 (1.06–1.41)	0.006	1.03 (0.87–1.22)	0.742
Place of residence				
Urban	1.00		1.00	
Rural	1.14 (0.97–1.35)	0.122	1.25 (1.04–1.49)	0.015

AOR, adjusted odds ratio, CI, confidence interval, COR, crude odds ratio.

## Discussion

The study aimed to assess fetal macrosomia prevalence in Bangladesh and its associated factors, revealing a prevalence of 11.6%, with higher maternal age group, higher education levels, history of physical attack, hypertension during pregnancy, male gender, and rural residence identified as significant determinants.

Various studies conducted in different regions have reported varying prevalence rates of fetal macrosomia. For instance, in China and Peru, the prevalence was found to be 7.4% and 7.5%, respectively (4, 22). However, there is a scarcity of data regarding nationwide prevalence rates of fetal macrosomia on a global scale. Hospital-based studies have provided insights into regional prevalence rates, with reports indicating rates of 9.4% in India and 7.3% in Pakistan, followed by some African countries where rates were recorded at 6.5% in Ethiopia and 2.3% in Tanzania (2, 7, 23–25). Furthermore, this study reveals that the prevalence of fetal macrosomia was notably higher among women with a history of gestational hypertension. Interestingly, the highest prevalence of fetal macrosomia was observed in the western part (Khulna division) of Bangladesh, potentially attributed to a higher occurrence of abdominal obesity in this population, which could influence post-pregnancy outcomes such as fetal macrosomia (10, 26).

The study revealed that higher maternal age group and higher levels of education among women were significant factors associated with fetal macrosomia (3, 18, 19, 25, 27–29). Women in higher age group is linked to an increased susceptibility to adverse maternal conditions like gestational diabetes mellitus and type 2 diabetes mellitus, both of which are known to be associated with fetal macrosomia (25, 30, 31). Furthermore, older women may be more prone to adopting sedentary lifestyles during pregnancy, which can lead to unfavorable outcomes such as gestational diabetes, excessive weight gain, and ultimately, macrosomia (24). Higher education is often associated with higher socioeconomic status, potentially leading to greater access to resources or increased consumption of energy-rich foods, resulting in higher maternal body mass index (BMI) and excessive weight gain during pregnancy, further increasing the risk of macrosomia (24, 32).

Remarkably, the study also found that women who experienced physical attacks were more likely to deliver macrosomic infants. Physical attacks have not been previously explored as a risk factor for macrosomia in the literature. However, physical or intimate partner violence has been associated with adverse birth or pregnancy outcomes in multiple studies conducted in several developing countries from Asia and Africa, including Ethiopia India and Nepal (33–36). Physical attacks on women can induce both physical and psychological stress, affecting the body's stress response system. This can result in elevated cortisol levels, reduced insulin sensitivity, and increased liver triglycerides, all of which contribute to the risk of gestational and type 2 diabetes mellitus, ultimately leading to fetal macrosomia. The findings suggest that achieving nationwide coverage of reproductive health education, care, and awareness, while incorporating family norms and practices, along with improving the quality and accessibility of antenatal care, could potentially help mitigate adverse pregnancy outcomes such as fetal macrosomia.

The findings indicate that mothers who experienced hypertension during pregnancy had a higher likelihood of delivering macrosomic infants compared to those without hypertension (37, 38). Previous studies in China and Iran have established a link between pregnancy-related hypertension and macrosomia (37, 39). Hypertension during pregnancy may arise from hormonal changes, including estrogen, progesterone, and relaxin, which affect the renin-angiotensin-aldosterone system (RAAS), leading to salt and water retention and increased plasma volume (40). As a result, pregnancy-related hypertension can lead to weight gain and insulin resistance during pregnancy, which in turn contribute to fetal macrosomia (41, 42).

The likelihood of fetal macrosomia, a condition characterized by excessive birth weight, was found to be higher among male infants. This observation aligns with previous research conducted in various regions. Studies from Asian countries, including Malaysia (18) and China (19), as well as those from the Middle East, such as Turkey (30), and African nations like Ethiopia (3) and Cameroon (43), have consistently reported a higher incidence of fetal macrosomia among male babies. One contributing factor to this phenomenon is the inherent biological differences between male and female infants. Genetically, male fetuses tend to exhibit slightly larger body weight, length, and head circumference compared to their female counterparts at the same gestational age (31, 44, 45). These differences in size can predispose male fetuses to an increased risk of macrosomia. Additionally, maternal behavior during pregnancy may play a role in the development of fetal macrosomia, particularly in pregnancies carrying male fetuses. Expectant mothers, upon learning they are carrying male babies, may inadvertently engage in excessive food consumption in an attempt to ensure a healthy outcome for their infants (39). This behavior can lead to disproportionate maternal weight gain and subsequently elevate the likelihood of fetal macrosomia (28, 45).

Furthermore, women residing in rural areas had a higher possibility of delivering macrosomic children compared to their counterparts. Women residing in rural areas had less access to proper dietary planning and prenatal care, resulting in abnormal weight gain during pregnancy (46). Moreover, rural women were more vulnerable to intimate partner violence, increasing the possibility of fetal macrosomia (34–36, 47). To reduce the occurrence of macrosomia, it is crucial to implement early screening, careful monitoring, and appropriate management strategies addressing the reproductive health of women. In addition, developing a local surveillance system collaborating with public and private healthcare providers might be effective for the management of adverse reproductive complications, like gestational hypertension. Promoting nutritional education and healthy lifestyle practices among women residing in rural areas might help to shrink the disparities in access to appropriate care and resources.

The study exhibits several notable strengths, notably its utilization of a nationally representative large sample size and the application of appropriate methodological frameworks. However, the investigation is not devoid of limitations. Principally, the present dataset may not faithfully encapsulate the entirety of the population of children under the age of five, owing to its reliance on a restricted dataset comprising children aged 2–3 years. Furthermore, the cross-sectional design of the study precludes the establishment of causal relationships between macrosomia and associated exposures. Moreover, the retrospective and self-reported nature of data collection introduces the possibility of underreporting, as well as information and recall biases. Several important mother's characteristics associated with macrosomia, such as gestational week or gestational age were not adjusted in the model due to their unavailability. Lastly, but not exhaustively, the study's scope is confined in terms of its generalizability to low- and middle-income countries.

## Conclusions

The prevalence of macrosomia among infants in Bangladesh stands at a noteworthy one in ten. This phenomenon has been linked to several significant determinants, including higher maternal age group, characterized by a higher level of education, a history of physical assault, hypertension during pregnancy, the birth of a male child, and residing in rural areas. Mitigating the escalating incidence of macrosomia demands a multifaceted strategy, encompassing enhancements in maternal nutrition, advocacy for healthy lifestyles, augmentation of access to quality prenatal care services, and the remediation of underlying socioeconomic, residential, and healthcare system hurdles. Moreover, the expansion of the study's purview necessitates further community-based investigations to glean comprehensive insights into this issue.

## Data Availability

The original contributions presented in the study are included in the article/Supplementary Material, further inquiries can be directed to the corresponding author.
