# Supplementary material for: Prevalence and determinants of fetal macrosomia in Bangladesh
Source: Front Pediatr. 2024 Aug 2;12:1405442. doi: 10.3389/fped.2024.1405442 (PMC11327860; doi:10.3389/fped.2024.1405442)
Supplement: Supplementary file 1 [file Table1.pdf]

SUPPLEMENTARY TABLE 1. Operational definitions of the independent variables

Variable	Description	Measurement	Scale of measurement
**Macrosomia (Outcome variable)**	**When a baby is born weighing more than 4000 grams (4 kg)**	**No, Yes**	Binary
**Mothers' age (in years)**	Age of mothers during data collection	15-19, 20-24, 25-29, 30-34, 35 and above	Discrete, categorical
**Level of education**	Educational status of the mother. Women who completed 1 to 5 class were referred as primary, 6 to 10th class secondary and from 11th to above were defined as higher secondary and above.	No formal education, Primary, Secondary, Higher secondary and above	Categorical
**Women experienced infant death**	Percentage of women age 15-49 years with a live birth in the last 2 years whose most recent live-born child was died after birth.	Yes, No	Binary
**Physical attack**	Percentage of women age 15-49 years who experienced physical violence or assault within the last 12 months	Yes, No	Binary
**Overall happiness**	Respondents were asked 'Taking all things together, would you say you are (1) very happy, (2) somewhat happy, (3) neither happy nor unhappy, (4) somewhat unhappy (5) very unhappy? Responses were further dichotomized as 'very happy or happy' *v.* all other responses (unhappy)	Happy, Unhappy	Binary
**Received antenatal care**	At least one medical surveillance and review performed during pregnancy for the early detection of possible complications of pregnancy.	No, Yes	Binary
**Had functional disability**	Women who had at least one difficulty following six domains (seeing, hearing, walking, cognition, self-care, and communication)	Yes, No	Binary
**Hypertension during pregnancy**	Women age 15-49 years who had high blood pressure during her last pregnancy.	No, Yes	Binary
**Tetanus toxoid injection during last pregnancy**	Women age 15-49 years with a live birth in the last 2 years who during the pregnancy of the most recent live birth were given at least two doses of tetanus toxoid containing vaccine or had received the appropriate number of doses with appropriate interval prior to the most recent birth.	No, Yes	Binary
**Children ever born**	Number of children who were born alive.	4 and higher, Less than 4	Binary
**Sex of newborn**	Different sex of the new born.	Male, Female	Binary
**Mass media exposure**	Mass media exposure through television, radio and newspaper/magazine has been defined as exposure to at least one media that exposes to at least once a week	No, Yes	Binary
**Wealth index**	Wealth index in the survey MICSis calculated, by the MICS authority, based on information on household characteristics and assets using principal component analysis. Then households are classified into quintiles based on the values of the wealth index, where households with	Poor, Middle, Rich	Categorical

	lower values of the index is considered as poorest and vice-versa		
**Place of residence**	Different residential areas across the country.	Urban, Rural	Binary
